# Aging transforms marine microplastics into reactive interfaces with environmental consequences

**DOI:** 10.1016/j.isci.2026.116102

**Published:** 2026-06-01

**Authors:** Chaoran Li, Lingchen Zhang, Junheng Li, Zhonglai Zhou, Zhihang Chen, Dayong Zhao, Fei He, Min Xu

**Affiliations:** 1 Jiangsu Key Laboratory of Ocean-Land Environmental Change and Ecological Construction, School of Marine Science and Engineering, Nanjing Normal University, Nanjing 210046, China; 2 Nanjing Institute of Environmental Sciences, Ministry of Ecology and Environment of the People’s Republic of China, Nanjing 210042, China; 3 Department of Brain Science, Imperial College London, UK/School of Biomedical Engineering and Imaging Sciences, King’s College London, London WC2R 2LS, UK; 4 Joint International Research Laboratory of Global Change and Water Cycle, The National Key Laboratory of Water Disaster Prevention, Hohai University, Nanjing 210098, China

**Keywords:** pollution, environmental toxicology, microbiofilms

## Abstract

Microplastics are pervasive contaminants in marine ecosystems and pose widespread threats to marine organisms and ecological processes. Diverse environmental stressors trigger microplastic aging through photooxidation, mechanical abrasion, and microbial colonization, which reshapes their physicochemical and biological traits. Aging induces polymer degradation, chemical group transformation, and increased surface roughness, greatly enhancing interfacial reactivity and adsorption toward heavy metals, organic pollutants, and antibiotics. It also alters microplastic transport, bioavailability, and toxicity, leading to cascading marine ecological risks. This review summarizes recent advances in marine microplastic aging mechanisms and surface evolution. An “interface reprogramming” framework is proposed to clarify how aging governs microplastic behaviors, pollutant interactions, and ecological fates. These findings deepen the understanding of microplastic hazards and support science-driven strategies for marine pollution mitigation.

## Graphical abstract

**Figure:**
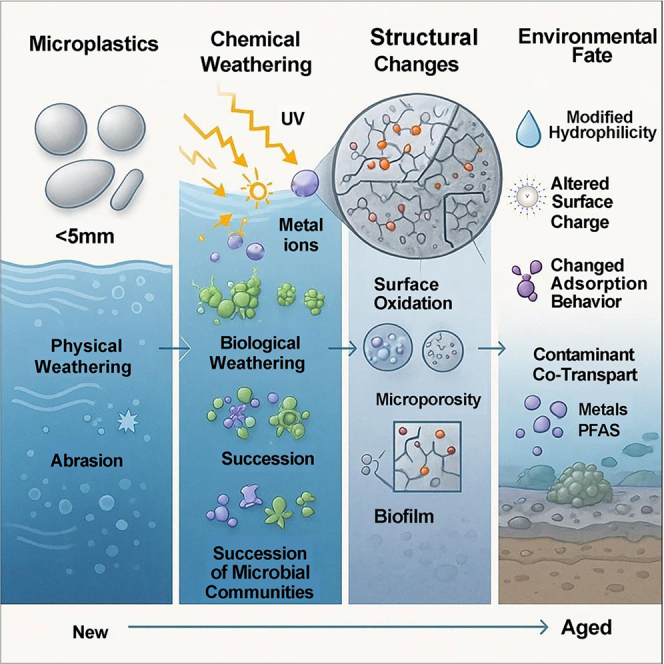

## Introduction

Microplastics are defined as polymer fragments or particles smaller than 5 mm. Due to their widespread distribution and persistence, they have recently attracted significant attention as ubiquitous contaminants in the global marine environment.^1^^,^^2^^,^^3^ Previous studies have demonstrated that the size and distribution of microplastics vary significantly across different marine compartments, impacting ecosystems from coastal sediments to marine zooplankton.^4^^,^^5^^,^^6^ Accordingly, the scope of this review focuses on the aging mechanisms and environmental fates of microplastics throughout the entire water column, rather than being limited solely to marine surface waters. Unlike traditional studies that primarily focus on the question of “whether microplastics exist,” current research emphasizes process-based scientific issues, such as how they change, why they change, and what consequences these changes bring. The term “aging” refers to the physical, chemical, structural, and surface characteristic transformations of microplastics under multiple environmental stresses. These stresses include light exposure, oxidation, mechanical wear, and biological attachment and subsequent microbial metabolism.^7^^,^^8^^,^^9^^,^^10^ For context, this aging process typically occurs over timescales ranging from days to months, depending on the environmental conditions and polymer type. Aging not only determines the integrity, mobility, and environmental fate of microplastics but also profoundly affects their interactions with metal ions, persistent organic pollutants, and biological entities in seawater. Therefore, systematically reviewing the aging mechanisms and surface property changes of microplastics in the marine environment is fundamental for understanding their environmental behavior, assessing ecological health risks, and guiding management strategies.^1^^,^^7^^,^^11^^,^^12^

From the perspective of sources and distribution, microplastics entering the marine system include both primary microbeads and industrial pellets, as well as secondary particles from the fragmentation of large plastic items. The sources span across urban runoff, domestic sewage, shipping, fisheries, coastal industries, and tourism, involving multiple sectors and links.^1^^,^^2^^,^^3^ Monitoring evidence shows that microplastics are nearly ubiquitous across all marine environmental media: from the nearshore intertidal zones to the open ocean surface, from the shallow to deep-sea sediments, and even in sea ice and polar ecosystems.^1^^,^^4^^,^^13^^,^^14^ It is important to note that the “presence” of microplastics in different spaces is just the starting point of the problem. What truly determines their environmental behavior is the aging process they undergo once they enter the marine environment, characterized by strong spatiotemporal heterogeneity. At the ocean’s surface, strong ultraviolet radiation and oxygen-rich conditions rapidly trigger photooxidative chain reactions. Simultaneously, wind and wave-induced mechanical fatigue cause surface breakdown. Furthermore, these processes are accompanied by the development of the “plastisphere,” a distinct ecological micro-niche formed through rapid microbial colonization and subsequent biofilm maturation on the plastic surface. These physical, chemical, and biological factors often act synergistically to alter the interfacial energy and diffusion boundary layer of the particles. This synergistic effect is specifically supported by a growing body of laboratory and field-based experimental evidence,^7^^,^^8^^,^^9^^,^^15^^,^^16^^,^^17^ rather than relying solely on model-based predictions. Consequently, this synergy results in significantly different apparent properties and environmental fates for the same polymer across different marine areas and seasons.

Fundamentally, the core material and interfacial changes driven by aging involve backbone fracture, crystallographic rearrangement, surface reconstruction, and functionalization. For example, ultraviolet-induced free radical reactions and subsequent oxidation lead to severe backbone cleavage. This process significantly reduces the molecular weight of polymers like polyethylene (PE), polypropylene (PP), and polystyrene (PS), with some studies reporting reductions in number-average molecular weight by 20% to over 50%.^7^^,^^10^^,^^15^ Simultaneously, these oxidation reactions generate oxygen-containing functional groups (e.g., carbonyl, hydroxyl, and carboxyl groups) on the surface. Furthermore, the preferential fracture and subsequent annealing of amorphous regions trigger an increase or reorganization in crystallinity. Depending on the polymer and environmental conditions, studies have reported crystallinity increases ranging from 10% to 30%.^7^^,^^9^ This structural reorganization alters mechanical brittleness, specific surface area, and pore structure. Correspondingly, surface functionalization intensifies, often indicated by a 0.5 to 2.0 increase in the carbonyl index. In terms of morphology, the appearance of cracks, grooves, and micropore networks significantly increases surface roughness. This structural degradation may expose areas enriched with fillers or additives, thereby forming chemical and physical “hotspot” adsorption sites. Ultimately, these surface and bulk structural reconstructions directly shape the interfacial potential of aged microplastics. On one hand, they enhance the adsorption capacity and selectivity for hydrophobic organic pollutants (e.g., chlorpyrifos) and emerging contaminants, such as specific per- and poly-fluoroalkyl substances (PFAS; e.g., PFOA and PFOS) and pharmaceutical residues (e.g., antibiotics).^15^^,^^18^ On the other hand, changes in surface charge and ζ potential strongly affect heterogeneous interactions with metal ions, fluorinated surfactants, and nanoparticles.^8^^,^^12^^,^^15^^,^^17^^,^^19^^,^^20^ Recently, the competitive adsorption and co-adsorption mechanisms of PFAS in seawater have also been discussed in the context of “naturally aged PS/PE,” providing new evidence for understanding these particles as active pollutant carriers.^20^^,^^21^

Despite this foundational understanding, current research faces significant methodological and extrapolation challenges.^2^^,^^3^^,^^22^ A primary challenge is the critical disparity between artificial and natural aging. Laboratory simulations typically employ steady-state, accelerated conditions—such as unnaturally high UV intensity, excessive oxidants, and constant agitation. In contrast, natural aging is a slower, non-steady-state process driven by complex, multifactorial stresses, including dynamic seawater chemistry and biofilm succession.^7^^,^^9^^,^^23^ Consequently, the functional group profiles and morphological structures of microplastics aged *in vitro* often deviate significantly from those found in the actual ocean. Furthermore, bridging the scale from micro-to nanometers remains a significant technical hurdle. Traditional characterization methods (e.g., routine FTIR, X-ray photoelectron spectroscopy [XPS], and SEM) face severe limitations in spatial resolution and quantitative consistency. Specifically, they struggle to capture the earliest stages of degradation, such as the initiation of nanoscale cracks and the formation of localized chemical gradients. Understanding these nanoscale interfacial dynamics is crucial, as they directly govern the micro-pulverization process and the subsequent formation of nanoplastics.^7^^,^^17^^,^^24^

Against this backdrop, a unified conceptual framework is urgently needed to address these methodological gaps. By leveraging fundamental principles of polymer science—traditionally applied to engineered materials—this review adopts a “time-stamped” perspective, tracing microplastics from their entry into the ocean through their aging processes. Rather than treating physical breakdown and chemical oxidation as isolated events, a logical framework centered on “interface reprogramming” is proposed. This framework systematically follows the chain from initial environmental drivers to structural responses, surface evolution, and ultimate ecological effects. Through a synthesis of recent studies, this review discusses the role of aging in pollutant co-transport, biofilm formation, and ecotoxicology, while providing an overview of characterization indices useful for cross-study comparisons. Ultimately, bridging the gap between the degradation kinetics of engineered materials and the environmental observations of marine microplastics provides a mechanistic basis for predicting their long-term fate and ecological risks at sea.

## Aging drivers of microplastics in marine environments

**Figure 1:**
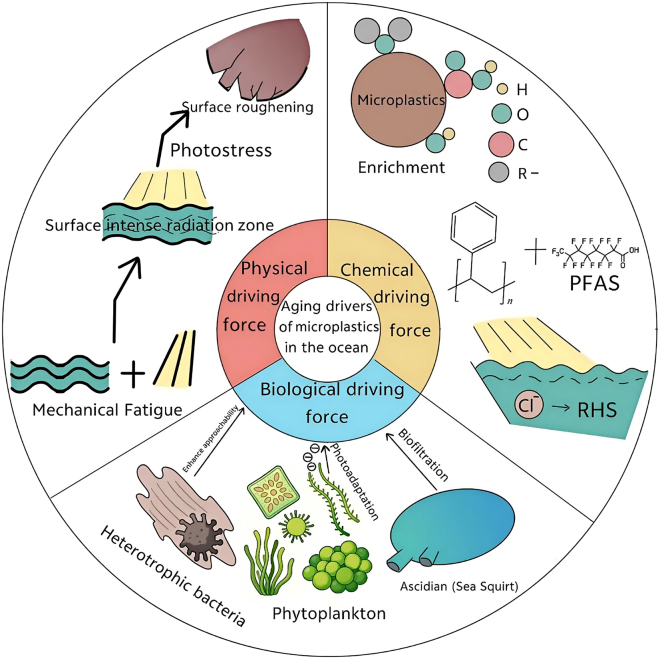
Microplastic aging drivers in marine environments

The aging of microplastics in the marine environment is driven by physical, chemical, and biological processes that interact with and amplify each other across multiple scales and interfaces (Figure 1). Below is a classified review of the key drivers.

### Physical drivers

From a physical perspective, mechanical fatigue induced by wave shear, tidal oscillations, and particle collisions, combined with photostress from high radiation areas, promotes crack initiation, pore expansion, and surface roughening. These effects significantly increase specific surface area and the density of reactive sites.^15^^,^^16^ During the bubble trapping and rupture process, particles can be rapidly transported and repeatedly exposed to gas-liquid-solid phase separations and rewetting, altering the near-surface dissolved oxygen, free radical flux, and adhesion dynamics, thereby reshaping aging pathways and rates.^17^ This “physical-transport-interface” interaction often serves as a precursor for subsequent chemical and biological processes, providing morphological and energetic conditions for functionalization and early biofilm colonization.^15^^,^^16^^,^^18^^,^^21^

### Chemical drivers

Chemical drivers include photolysis, oxidation, and the synergistic effects of salt ions/metals. Gao and colleagues systematically summarized experimental and field evidence of free radical chain reactions triggered by ultraviolet radiation, visible light, and dissolved oxygen. They pointed out that the accumulation of oxygen-containing functional groups such as carbonyl, carboxyl, and hydroxyl groups on the surface is a direct mechanism for changes in hydrophilicity, charge, and adsorption selectivity after aging.^7^ Barhoumi and others emphasized that natural aging of polystyrene in seawater significantly enhanced its adsorption capacity for both traditional and emerging PFAS, suggesting the universality of the “aging-interface contamination” interaction.^18^ In natural seawater, dissolved organic matter (DOM) and trace metal ions directly modulate the photochemical aging of microplastics. Experimental evidence demonstrates that DOM acts as a natural photosensitizer; under UV irradiation, it generates reactive oxygen species (ROS) such as hydroxyl radicals and singlet oxygen, which accelerate polymer chain scission and surface oxidation.^7^^,^^9^ Concurrently, trace transition metals (e.g., iron) can catalyze photo-Fenton-like reactions, further amplifying the ROS flux at the plastic-water interface. These experimentally validated DOM/metal interactions not only accelerate the structural degradation of microplastics but also significantly alter the adsorption-desorption dynamics of co-existing pollutants.^7^^,^^25^ In coastal environments with strong light and high salinity, halide ions, especially Cl^−^, can participate in photochemical generation of active halogen species, driving the photooxidation and chain scission of materials like PP.^26^ Vertical light fields and variations in salinity-temperature structures further shape spatial heterogeneity in aging rates through irradiation dose and oxygen supply constraints.^19^^,^^27^

### Biological drivers

Biological drivers involve both community-level processes such as “filtering, enriching, and redistributing,” and microbial-level processes like “attachment, metabolism, and mineralization.” Harel, Zucker, and Shenkar discovered that the biological filtration by sea squirts could significantly alter the microplastic composition and particle size structure in the water column, suggesting that filter-feeding invertebrates reshape the “exposed-reactive” particle pool through selective ingestion and excretion.^28^ For example, Plummer, Garcia, and Diaz demonstrated that phytoplankton can release extracellular superoxide anions (O_2_^−^·) as a light adaptation strategy, thereby increasing local ROS levels.^29^ These photochemically or biologically generated ROS (e.g., hydroxyl radicals, superoxide) are highly reactive and can directly attack polymer backbones, initiating chain scission and leading to the formation of oxygen-containing functional groups on the particle surface, driving surface oxidation and additive leaching of adjacent particles. The attachment and biofilm formation of heterotrophic bacteria change the hydrophobicity-hydrophilicity pattern, surface potential, and diffusion boundary layer thickness, significantly enhancing interface affinity for metal ions and polar organic compounds, and mediating chain scission and depolymerization through extracellular enzyme systems.^8^^,^^9^^,^^10^ Mori-Bazzano and others found that depth, seasonality, and bacterial community dynamics could significantly affect plastic degradation and aging trajectories in semi-enclosed lake systems,^30^ providing analogous evidence for seasonal differences in coastal semi-enclosed bays, lagoons, and estuaries.

The specific reactivity of different ROS species toward various polymer backbones is a critical yet understudied aspect of biologically driven aging. To mechanically link biogenic ROS production to measurable polymer oxidation, a classic radical auto-oxidation pathway must be considered. In seawater containing trace transition metals (e.g., Fe or Cu), phytoplankton-derived superoxide (O_2_^−^·) and its disproportionation product, hydrogen peroxide, can undergo Fenton-like reactions to generate highly non-selective hydroxyl radicals (·OH).^29^^,^^31^ With a high oxidation potential (E° = 2.80 V), ·OH initiates oxidation by abstracting labile hydrogen atoms from the microplastic backbone (e.g., tertiary carbons in PP or secondary carbons in PE), forming carbon-centered macroradicals (P·).^32^ These macroradicals rapidly react with dissolved oxygen to form peroxy radicals (POO·) and subsequently hydroperoxides (POOH). The eventual cleavage of these unstable intermediates results in polymer chain scission and the accumulation of terminal carbonyl (C=O) and hydroxyl (–OH) groups. Analytically, this biologically driven oxidation becomes directly measurable through a quantifiable increase in the carbonyl index (CI) (via FTIR at ∼1,710–1,730 cm^−1^), an elevated surface O/C atomic ratio (via XPS), and a distinct downward shift in molecular weight distribution (MWD) (via gel permeation chromatography [GPC]).^7^^,^^9^^,^^10^ In contrast to the non-selective ·OH, superoxide (O_2_^−^·) and singlet oxygen (^1^O_2_) are more selective, preferentially attacking unsaturated regions or functional groups, such as those found in polymers with aromatic rings (e.g., PS) or carbonyl groups introduced during initial oxidation.^7^^,^^9^^,^^32^ During seasonal phytoplankton blooms, the dominant ROS species produced can shift—for example, superoxide release is common in many algal species,^29^ while conditions of high light and nutrient limitation can promote ·OH generation via photochemical pathways involving DOM.^33^ This seasonal variation in ROS profiles could lead to different aging pathways and rates, with polyolefins potentially more susceptible during ·OH-dominated periods and aromatic polymers more vulnerable when ^1^O_2_ or superoxide fluxes are high.^30^ Understanding these molecular-level preferences and their temporal dynamics is essential for predicting the fate of different polymer types in productive marine ecosystems. Furthermore, these aging-induced structural defects and functional groups provide energetic sites that stabilize environmentally persistent free radicals (EPFRs), which can further catalyze persistent ROS production at the interface, creating a self-sustaining degradation loop.^32^

### Coupling effects of multi-drivers

The coupling across processes often amplifies aging effects and alters trajectory pathways. Research by Xiang and others on bubble-particle interactions revealed a “bubble transportation-interface enhancement-re-sedimentation” cycle that rapidly exchanges the surface light/oxygen environment with the bottom weak light/low oxygen environment, thus accelerating aging effects over time and expanding them spatially.^17^ Both engineered and natural particles can also participate in coupling interactions: magnetic biochar, colloidal minerals, and biogenic debris act as intermediaries for light/heat/electrons in turbid waters, altering the local reaction field and microplastic surface charge environment. In oil spill events, Dong and others demonstrated that the synergy between biological and physical particles could accelerate hydrocarbon degradation, indirectly altering the microplastic interface microenvironment and adsorption competition patterns.^34^ Under advanced oxidation conditions, Dang and colleagues discovered that sulfate radicals and hydroxyl radicals produced in a UV/persulfate (UV/PDS) system can drastically reshape interface chemistry under complex conditions involving DOM, salt, and metals.^33^ It must be emphasized that UV/PDS is an engineered water treatment system, not a natural open-ocean condition. However, discussing such engineered systems is highly relevant to marine environmental assessments because advanced wastewater treatment facilities serve as major point sources of marine microplastics.^1^^,^^3^ Microplastics passing through these aggressive oxidative or biological treatment facilities undergo severe “pre-aging” prior to their discharge into coastal waters.^9^^,^^35^ Consequently, they enter the marine environment not as pristine, inert polymers, but as highly oxidized, surface-activated particles. Once discharged into the ocean, these pre-aged plastics exhibit significantly accelerated degradation kinetics and altered pollutant-binding capacities compared to particles aged entirely in nature.^7^^,^^36^ When such pre-activated particles enter marine reaction fields, the synergy between their engineered pre-aging and natural weathering promotes faster migration of additives and creates distinct toxicological profiles.^10^^,^^12^

To reduce the predictive uncertainties inherent in current aging studies, simulation protocols must transition from static intensification to iterative calibration against *in situ* observations.^7^^,^^36^ One primary optimization strategy involves prioritizing spectral and irradiance fidelity. Most laboratory setups utilize high-intensity UV lamps that neglect the specific spectral power distribution of the actual marine light field. Optimization should focus on utilizing LED-based solar simulators that replicate the specific UVB to UVA ratios found at varying ocean depths.^19^^,^^27^ By introducing scaling factors based on radiometer data, researchers can ensure that the cumulative photon dose in the laboratory represents a realistic environmental exposure time.

Another essential strategy is the integration of biogenic-chemical coupling into oxidative setups. Traditional chemical-only aging lacks the micro-niche effects of the plastisphere. Optimization involves the use of hybrid incubation protocols, where microplastics are first colonized by natural marine biofilms *in situ* or in mesocosms before being subjected to controlled stressors. This captures the role of extracellular polymeric substance (EPS) in trapping ROS and regulating the diffusion boundary layer, which fundamentally alters surface functionalization patterns.^9^^,^^37^

Furthermore, the adoption of iterative feedback loops is essential for model validation. Laboratory-aged particles should be directly benchmarked against field-retrieved samples using high-resolution indicators such as the CI and MWD.^7^^,^^17^^,^^24^ If the laboratory aging trajectory deviates from the field aging fingerprint, simulation parameters, including temperature, salinity, or oxidant flux, must be iteratively adjusted until the chemical and morphological failure modes align.^7^^,^^36^ This transition from blind acceleration to calibrated simulation is critical for transforming aging studies into reliable inputs for global plastic fate models.

### Material-specific aging characteristics

On the material level, there is “material specificity” in aging pathways for conventional polymers, new elastomers, and degradable polyesters. The fundamental divergence lies in the polymer backbone chemistry: conventional polyolefins like PE and PP possess carbon-carbon backbones, whereas bio-based polyesters such as polylactic acid (PLA) and polybutylene adipate terephthalate (PBAT) contain hydrolytically susceptible ester bonds. For polyolefins, aging is primarily governed by photo-oxidative pathways in sunlit surface waters, where UV radiation initiates free radical chain reactions leading to backbone cleavage and CI increase.^7^^,^^10^ In contrast, in aphotic and anaerobic environments like deep-sea sediments, these conventional microplastics exhibit extreme persistence due to the absence of light and molecular oxygen required for oxidative degradation.^9^^,^^19^

Furthermore, the degradation of PLA and PBAT is dominated by hydrolytic pathways, which can proceed independently of UV exposure. In marine sediments, water molecules directly attack the ester linkages, causing bulk or surface erosion that significantly broadens the MWD.^38^^,^^39^ This is supported by Tseng and others, who found that the fungus Purpureocillium lilacinum could significantly enhance the biodegradation flux of PBAT and alter MWD and end-group composition through a multi-enzyme system.^38^ While polyolefins remain nearly inert in anaerobic niches, these degradable polyesters undergo accelerated microbial fermentation and enzymatic hydrolysis.^40^ Similarly, Zhao and colleagues studied the mechanism of foamed thermoplastic polyurethane (ETPU) and showed that phase separation of soft and hard segments, addition/depolymerization balance, and crosslinking side reactions collectively determine mechanical and chemical tolerance.^41^

These findings resonate with the biological film-chemical aging synergy in seawater systems, suggesting that the “material chemistry-microbial ecology-environmental factors” triadic coupling is the key to understanding aging heterogeneity.^8^ The aging drivers in the marine environment are not a simple linear accumulation of processes but are controlled by nonlinear coupling among factors such as light field, fluid dynamics, salinity/metal ions, DOM, microbial composition, and particle coexistents. The result manifests as accelerated surface functionalization and roughening, migration of hydrophilic/charged characteristics, reconfiguration of adsorption sites, and biofilm-mediated interface “reprogramming,” which further influences pollutant co-transport, particle settling and resuspension, as well as long-range transport fluxes and risk externalities.^7^^,^^8^^,^^9^^,^^10^^,^^17^^,^^18^^,^^19^^,^^21^^,^^23^^,^^26^

### Quantitative kinetics of microplastic aging

To transition from a narrative description to a data-driven framework, the “time-series perspective” of aging must be grounded in quantitative kinetics. The degradation of microplastics is generally modeled as a pseudo-first-order reaction, where the rate of functional group accumulation or mass loss is expressed as

$$\frac{d\left[P\right]}{dt}=-k\left[P\right]$$

where [*P*] represents the concentration of pristine polymer sites and *k* is the apparent degradation rate constant.

In the sunlit marine surface microlayer, *k* values for microplastics (e.g., PE, PP) are primarily driven by the UV intensity (*I*) and temperature (*T*), often following an Arrhenius-like dependence: *k* = *A*·*I*^*n*^·*exp*(-*E*_*a*_/*RT*). Reported values for the CI increase rates in surface waters suggest *k*_*CI*_ ranges from 0.01 to 0.05 *day*^s−1^ under peak summer irradiance.^5^^,^^18^^,^^21^ In contrast, in the aphotic water column and benthic sediments, where *I* ≈ 0, aging transitions to a biologically mediated process with significantly lower *k* values (10^−3^ to 10^−4^ *day*^−1^), primarily governed by the secretion rate of extracellular enzymes and oxygen availability.^9^^,^^30^

The environmental half-life (*T*_1/2_) of microplastics thus exhibits extreme spatial heterogeneity. For a typical 100 *μm* PE particle, *T*_1/2_ may range from months in the Mediterranean surface to centuries in the deep-sea trenches. Quantifying these *k* values across different “marine reaction fields” is essential for moving toward predictive modeling of nanoplastic formation and pollutant co-transport fluxes.

## Polymer structural and physicochemical changes during aging

In marine environments, microplastic aging is dominated by coupled structural and surface changes: polymer chain scission, crystallographic rearrangement, surface functionalization, and network reconstruction. These changes ultimately impact the mechanical, thermal, and interfacial behaviors of the particles.^10^ The primary step in this chain is the chemical degradation of the main and side chains. This degradation is driven by the combined action of light, oxygen, and metal ions, along with the active involvement of microbial biofilms functioning as reactive biological membranes. These biological membranes participate directly by secreting extracellular enzymes (such as oxidoreductases and hydrolases) that attack specific polymer bonds. Furthermore, the EPSs within these membranes create localized, acidic, or highly oxidative micro-niches that trap ROS and significantly accelerate degradation kinetics.^8^^,^^9^^,^^37^ Consequently, polymers undergo chain scission, depolymerization, and the introduction of functional groups. This leads to a downward shift and broadening of the MWD, which further alters the viscoelastic spectrum and failure modes.^7^^,^^10^^,^^18^^,^^21^

Evidence from biodegradation and chemical aging shows that the selective cleavage of peptide/amide bonds or hydrocarbon chains is the main cause of molecular weight reduction. For example, Yu et al. isolated *Alcaligenes faecalis* EPDB-5 from oilfield sludge, revealing the enzymatic chain scission pathways and functional group reconstruction of hydrolyzed polyacrylamide (HPAM).^42^ GPC clearly showed significant molecular weight reduction and low-molecular-weight segment enrichment. While HPAM from oilfield sludge is an industrial polymer rather than a common hydrophobic marine plastic (like PE or PP), its degradation serves as a crucial mechanistic model. Specifically, it demonstrates how bacterial extracellular enzymes achieve carbon-backbone scission and nitrogen-group transformation.^42^ This enzymatic model is highly relevant to marine environments, as it conceptually explains the biodegradation pathways of marine polyamides (PAs, such as nylon fishing nets) and provides fundamental insights into how the plastisphere community might metabolize the carbon backbones of weathered polyolefins once they become sufficiently oxidized and hydrophilic.^8^^,^^9^^,^^10^

Aging not only affects chemical bonds but also reshapes crystallographic structures and phase proportions. Zeng et al. showed that the amorphous regions are primarily damaged, inducing heterogeneous recrystallization, which leads to changes in grain size distribution and sheet thickness, thereby shifting the melting point, glass transition temperature, and thermal decomposition onset temperature.^43^ In biodegradable polyester systems, Zhang et al. achieved significant improvements in thermal and mechanical stability by co-regulating the crystal structure of PLA fibers through long-chain branching and drawing.^39^ This “structural engineering” also suggests that the initial crystallization/defect state of materials will determine their subsequent sensitivity to seawater aging. As demonstrated by Leite-Barbosa et al., for plastically modified or plant oil-containing polyolefins, after aging, the migration, phase separation, and secondary oxidation of the plasticizer phase alter the interactions between crystal layers, ultimately resulting in the time decay of thermal stability and dimensional stability.^44^

Aging often leads to a transition from ductile-tough fracture to brittle-quasi-cleavage. Zarzyka et al. demonstrated in polyhydroxybutyrate (PHB) and linear polyurethane/organic nanofiller composites that network structures and filler interfaces could temporarily enhance modulus and strength, but under UV/oxidation, interfacial debonding and accumulated network fatigue cause a “hysteretic” strength decline.^45^ Molecular dynamics results on hydrogen-bonded polymers showed that the number and strength of hydrogen bonds jointly determine energy dissipation and crack propagation paths. Aging weakens the hydrogen-bond network, making the system more prone to microcrack-induced instability.^46^ In biomedically crosslinked materials, Zhang et al. showed that crosslinking architecture (density/topology) regulates energy dissipation in ligamentization and suture retention, suggesting an interlinking, aging and toughness engineering window.^47^

Surface charge and the point of zero charge (PZC) are crucial in determining adsorption/desorption and colloidal stability. Leffler et al. revealed that the PZC of nanoscale anatase TiO_2_ shifts with decreasing particle size and is related to surface defect density and hydroxylation degree.^48^ Surface charge and PZC are crucial in determining adsorption/desorption and colloidal stability. Leffler et al. revealed that the PZC of nanoscale anatase TiO_2_ shifts with decreasing particle size, a phenomenon driven by surface defect density and hydroxylation degree.^48^ While this finding is specific to metal oxides, it provides a valuable mechanistic analogy rather than a direct equivalence for aged microplastics. In engineered plastics, TiO_2_ is frequently used as a whitening pigment or UV stabilizer. During aging, the polymer matrix degrades and exposes these inorganic fillers, creating a “composite interface.” On such surfaces, the observed PZC is a synergistic result of the polymer’s organic functional groups (e.g., carboxyls) and the inorganic filler’s hydroxylated sites.^48^^,^^49^ Therefore, while the PZC shifts of TiO_2_ and polymers are governed by different chemical species, the reconstruction of a hydroxyl-enriched surface on aged microplastics, whether through polymer oxidation or filler exposure, leads to a predictable evolution of the ζ potential and electrical double-layer structure. This evolution ultimately dictates the particle’s heterogeneous interactions with metal ions, polar pollutants, and DOM.^7^^,^^24^ The behavior of nitrogen-containing PAs in sulfur-containing environments also requires consideration. Lazari et al. found that exposure to H_2_S scavengers altered the microscopic kinetics and hydrogen-bond network of PA, suggesting that surface charge and diffusion properties of PA microplastics in hypoxic/reductive marine areas (such as cold springs) might differ from conventional scenarios.^50^

Under visible light conditions, Sura and Nain confirmed that GO/SCN composites could synergistically promote the degradation of low-density polyethylene (LDPE) films and BPA, pointing to a possible linkage of environmental catalysis, surface activation and chain scission.^51^ Although this strategy is closer to engineering scenarios, its insights into free radical flux and surface energy state regulation align methodologically with the reactive environments formed by metal/mineral particles and extracellular ROS in nature.^29^^,^^33^ From a macro-micro correlation perspective, Xi et al. established a relationship between macroscopic properties and molecular mechanisms during UV aging using asphalt as a model system, providing ideas for the cross-scale coupling of “structure-performance-aging mechanisms” in polymer materials, which can be applied to the mechanical characterization and lifespan evaluation of microplastics.^52^

## Evolution of surface properties and their environmental significance

**Figure 2:**
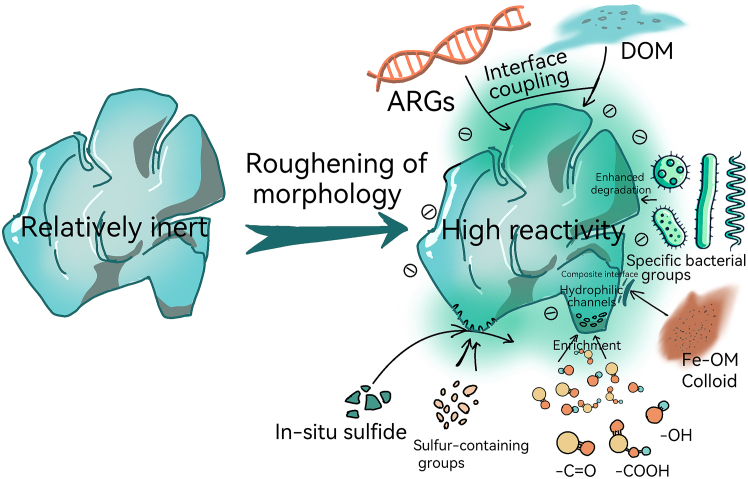
Surface property evolution and environmental significance

While engineered materials are often functionalized for specific industrial purposes, environmental aging acts as a “stochastic surface engineering” process. The proliferation of oxygen-containing functional groups, a common failure mode in structural polymers, transforms aged microplastics into reactive ion-exchangers in seawater, explaining their increased affinity for heavy metals and PFAS compared to pristine materials. The evolution of microplastic surface properties is synergistically driven by multiple mechanisms closely linked to the aforementioned structural and chemical changes. Aging in seawater environments drives microplastics from a “relatively inert” to a “highly reactive” state. Processes such as roughening of morphology, proliferation of oxygen/sulfur-containing functional groups, migration of surface energy and charge, biofilm coverage, and leaching of substances intertwine, determining the migration of particles in porous media, their fate at water-sediment interfaces, and their interactions with metal ions, antibiotics-resistant genes (ARGs), and DOM (Figure 2). Wang et al. showed that once reduced PBAT microplastics undergo a “rebound” in surface energy and roughness, their mobility is significantly amplified, suggesting a direct link between aging, surface energy and mobility.^53^ Mechanistically, this “rebound” is driven by the synergistic effects of chemical reduction and polymer chain relaxation. The initial reduction process removes surface-passivating layers, exposing fresh ester and carbonyl groups. Subsequently, the high-energy environment facilitates the migration of polar segments from the bulk to the surface to minimize interfacial Gibbs free energy. This re-orientation, combined with the emergence of new oxygen-containing functional groups during the recovery phase, creates a more chemically active and physically heterogeneous surface, leading to the observed increase in both surface energy and micro-topographic roughness.^53^^,^^54^ Meanwhile, Wang et al. found that photoaging leaches out DOM components with different molecular properties from microplastics, which not only alters the particles’ surface chemistry but also reshapes the “reactive” organic carbon pool in the water.^55^ In the microbiological context, Tang et al.’s review shows that aging, through functionalization and biofilm mediation, enhances the adsorption and transport potential of ARGs, providing a mechanistic basis for the microplastics and ARGs triad risk coupling.^56^ Additionally, the synergistic effects of chemical aging and biofilms enhance metal ion adsorption (such as copper), which was verified in the PA and PLA systems.^8^

From morphology and porosity, the formation of cracks, grooves, and micropores is usually accompanied by increased specific surface area and surface energy, facilitating heterogeneous adhesion and nucleation.^53^ Under a sulfide background, Kim et al. revealed that “*in situ* sulfides” can mediate rapid aging on polystyrene surfaces and introduce sulfur-containing groups, which subsequently alter the fate of heavy metals at the interface, pointing to a specific aging pathway in reductive/anaerobic environments.^57^ Extreme accidents offer “natural experiments” with multi-stress coupling: Jayasekara et al. conducted environmental forensics on marine microplastics released during the X-Press Pearl incident, recording structural changes driven by heat, chemical, and mechanical wear (including voids and deep cracks),^58^ confirming the coupling rhythm of morphological degradation and functional group formation. Different aging pathways feedback into adsorption capabilities: Liu et al. systematically compared and found significant differences in the effects of various aging methods on heavy metal adsorption^16^; Zhang et al. confirmed that under stormwater runoff conditions, the load of heavy metals and the surface state of microplastics interact, influencing ecological risk thresholds and exposure windows.^59^ It is important to note that artificial weathering protocols may alter conclusions regarding subsequent biodegradation and interfacial behavior, indicating that methodological choices themselves are implicit variables in “surface property evolution.”^36^

On the surface chemistry level, the accumulation of carbonyl, hydroxyl, and carboxyl groups is the most intuitive spectroscopic fingerprint of aging, directly leading to enhanced hydrophilicity, migration of ζ potential, and restructuring of hydrogen bond networks. Kanu et al. showed through thermodynamic decomposition that the adsorption of molecules on microplastic surfaces has distinct enthalpy-entropy contributions, and changes in functional groups and surface ordering rearrange the balance of adsorption driving forces, explaining the “why aging makes surfaces stickier/selective” interfacial phenomena.^54^ Furthermore, Zhou et al. demonstrated in another system that surface hydroxyl groups effectively promote interfacial electron/proton transfer and reaction kinetics.^49^ This “hydroxyl-enhanced interfacial reactivity” rule can also be applied to understand the increased surface chemical activity of aged microplastics in redox environments. Complementing this, nanocellulose obtained through hydrolysis in green solvents is rich in chelating/hydrogen bonding sites,^60^ while Ruiz-Caldas et al.’s cationic/anionic cellulose nanofibers offer a reference for “programmable surface charge;”^61^ when these charged biobased colloids coexist with aged microplastics, they can form two distinct pathways: charge bridge aggregation-settling or heterogeneous composite-cotransport, depending on ionic strength and DOM competition for surface sites.

Biofilms and microbial processing further “reprogram” surface properties. Newrick et al. showed that specific bacterial communities can enhance the biodegradation efficiency of high-density polyethylene (HDPE) microplastics^62^; Ma et al. revealed that microorganisms can erode hydrocarbon polymer structures through metabolic networks in asphalt systems,^63^ which also holds relevance for hydrocarbon polymer microplastics in seawater. Meanwhile, differences between artificial weathering and natural aging alter subsequent microbial community’s synergistic degradation capacity and interfacial chemical readout.^36^ In environmental chemistry, Wei et al. pointed out that iron-organic matter (Fe-OM) colloids play a key role in metal migration,^64^ implying that in Fe-OM-rich nearshore/estuarine waters, aged microplastics, not only as “passive carriers,” may form “composite interfaces” with Fe-OM colloids that jointly regulate metal mobilization and re-deposition. Functionalized carbon materials in environmental media can also alter local reaction fields: Cao et al.’s N, S-doped carbon dots show significant corrosion inhibition and interfacial regulation capabilities,^65^ while Cai et al.’s sulfur-doped porous lignin-carbon exhibit high capacity performance in water treatment.^66^ The environmental significance of these engineered carbons lies in their “active coordination” with aged microplastic surfaces. Specifically, the pyridinic nitrogen and thiophenic sulfur sites on these carbon materials act as strong Lewis bases. They can undergo site-specific coordination or hydrogen bonding with the electron-deficient carbonyl groups and hydroxyl sites on aged microplastics. This interaction creates a “heterogeneous coating” that reconfigures the particle’s interfacial potential, potentially masking or amplifying its original adsorption capacity for heavy metals and organic pollutants through complexation-bridging mechanisms.^49^^,^^65^^,^^66^

From an environmental significance perspective, aging-induced surface changes impact the marine system through four distinct pathways. First, direct ecological effects occur as chain scission promotes the micro-pulverization of particles into the nanoplastic scale, altering their bioavailability and enabling transmembrane transport.^7^^,^^67^ Second, pollutant co-transport is intensified by surface reconstruction. The accumulation of carbonyl and hydroxyl groups, alongside the leaching of microplastic-derived DOM, reconfigures the interfacial potential to enhance the uptake of metals and antibiotics.^54^^,^^55^ Third, biological interaction is redefined by the mature “plastisphere.” Biofilm-coated aged microplastics act as mobile ecological niches that facilitate the enrichment and horizontal gene transfer of ARGs.^56^^,^^68^ Fourth, ecosystem disturbances arise during extreme events, such as the X-Press Pearl incident. Highly activated particles with deep cracks provide a pulse of high-density adsorption sites and chemical leaching, potentially shifting local redox conditions and material cycles.^58^^,^^69^

To avoid speculative assumptions regarding chemical interactions, the specific role of sulfur-containing groups in anaerobic or sulfidic environments must be mechanistically defined. On sulfidized polystyrene surfaces, these sulfur groups act as “soft” Lewis bases according to the hard-soft acid-base (HSAB) theory. They exhibit a high polarizability that allows for strong, covalent-like coordination with “soft” heavy metal cations, such as Hg^2+^, Cd^2+^, and Pb^2+^.^57^^,^^66^ Mechanistically, this involves the substitution of weaker hydration shells with stable metal-sulfur (M-S) complexes, which can alter the metal’s valence state and coordination geometry. Such site-specific interactions fundamentally change the thermodynamic stability and remobilization potential of heavy metals at the water-sediment interface, distinguishing aged microplastics from pristine particles that rely primarily on weaker electrostatic or van der Waals forces.^49^^,^^57^

The diversity of environmental contexts further dictates the rate of this surface evolution. Ren et al. found strong medium dependence in polyvinyl chloride (PVC) aging across different soil types,^70^ while the sensitivity of heavy metal adsorption to specific “aging methods” suggests that surface activity is highly context-dependent.^17^ Therefore, cross-study comparisons must transition from describing generic “high adsorption” to reporting standardized, reusable indicators. These include the CI, PZC, specific surface area, and the molecular characteristics of leached DOM. Integrating these comparable indicators with contextual reaction field parameters—such as salinity, ROS hotspots, and biofilm time series—will transform “surface evolution” from a descriptive observation into a quantifiable variable for predictive risk assessment.^36^^,^^53^^,^^64^^,^^71^

## Environmental behavior and effects of aged microplastics

Aging redefines microplastics as “active interfaces” that exert complex effects on pollutant adsorption-desorption, biofilm formation, and nutrient cycling, as well as on cross-boundary transport. Based on soil systems, Gong et al. showed that water content reshapes the surface energy and colloidal stability of biodegradable microplastics by influencing functionalization and pore structure, thus altering their interactions with the matrix and migration capacity.^40^ In marine environments, this surface evolution and environmental behavior coupling is often more pronounced: Gao et al.’s review indicated that photooxidation and mechanical stress cause cracks, micropores, and enrichment of oxygen-containing functional groups on PP, which enhances its affinity for organic pollutants and metal ions, thus changing its colloidal stability and sedimentation pathways.^7^

Therefore, the environmental behaviors and effects of different types of microplastics across various studies, including their size ranges, methods, and key findings, have been summarized in the following Table 1.

**Table 1:** Microplastic environmental behavior and effects analysis

Polymer Type	Polymer category	Size range	Key aging factor	Environmental behavior	Ecological effect	Reference
PP	Polyolefin	10–500 μm	Photooxidation, mechanical stress	Surface cracking, micropore formation, oxygen-containing functional groups	Enhanced affinity for organic pollutants and metals	Gao et al.^7^
PS	Polyolefin	0.1–10 μm	Bubble-mediated transport	Bubble carriage-interface aggregationre-sedimentation cycle	Accelerated adsorption/desorption, amplified co-transport	Xiang et al.^17^
PS	Polyolefin	5–200 μm	Natural aging in seawater	Surface roughening, increased polar sites, early biofilm formation	Enhanced PFAS adsorption capacity	Barhoumi et al.^18^
PET	Polyester	0.001–1 μm	Fe-NOM coupling	Forms “composite interfaces” with Fe-NOM colloids	Regulates metal fate at sediment-water interfaces	Li et al.^25^
PET	Polyester	20–300 μm	Biofilm formation	EPS provides complexation and hydrogen bonding sites	Altered adsorption isotherms of POPs and metals	Mishra et al.^37^
PET	Polyester	10–200 μm	Aging-induced functionalization	Enhanced surface roughness and functional groups promote biofilm adhesion	Increased enrichment and transport of antibiotics and ARGs	Tang et al.^56^
PVC	Vinyl polymer	0–100 nm	UV aging	Carrier effect co-acts with mercury	Enhanced mercury toxicity to copepods	Xie et al.^67^
PVC	Vinyl polymer	10–250 μm	Biofilm succession	Dynamic adjustment of interface affinity and diffusion boundary layer	Prolonged residence time at water-sediment interfaces	Zhou et al.^72^
PVC	Vinyl polymer	5–180 μm	Biofilm formation	Formation of micro-niches on plastic surfaces	Acts as “mobile nodes” for ARGs and resistant strains	Pal et al.^73^

Findings from Barhoumi et al. indicate that natural aging of PS in seawater enhances its adsorption of emergency and traditional PFAS, attributed to surface roughening, increased polar sites, and early biofilm involvement.^18^ These studies collectively demonstrate that aging-mediated surface roughening and functionalization are key factors in enhancing the carrier capacity of microplastics for a wide range of contaminants. Mishra et al. systematically reviewed microplastic, biofilm and pollutant complexes, showing that EPS on the surface of polyethylene terephthalate (PET) provide complexation and hydrogen bonding sites, altering the adsorption isotherms and kinetics of persistent organic pollutants and metals.^37^ Zhou et al. further emphasized that biofilm succession on PVC dynamically adjusts the interface affinity and thickness of the diffusion boundary layer, significantly prolonging particle residence time at water-sediment and biological surfaces.^72^ From the perspective of bubble-mediated cross-boundary transport, Xiang et al. revealed the bubble-carried, interface aggregation and re-sedimentation cycle at the nanoscale for PS, which accelerates adsorption/desorption processes and amplifies the range of co-transported pollutants.^17^ Su et al. confirmed in the microalgae and microplastic coupled system that floc formation in PP altered the effective density and sedimentation flux of particles, changing the exposure window of co-adsorbed pollutants.^74^

Beyond chemical transport, aging-induced functionalization promotes biofilm adhesion and the subsequent enrichment of antibiotics and ARGs.^56^ Pal et al. highlighted that micro-ecological niches on aged plastic surfaces become “mobile nodes” for resistant strains,^73^ while Balta et al. suggested that synergistic effects under multi-pollutant pressure could amplify resistance transmission.^75^ These results collectively point to the fact that aged microplastics, through surface and biofilm “reprogramming,” alter microbial community structure and functional networks.

Regarding toxicity, Xie et al. suggested that UV-aged PVC nanoparticles significantly increase mercury toxicity to copepods under multi-generation exposure, demonstrating a “carrier + stress amplification” synergy.^67^ To move beyond speculative claims, this amplified toxicity must be supported by comparative toxicological evidence. Recent studies demonstrate that while pristine particles cause limited physical irritation, aged microplastics, due to their higher surface energy and smaller size, induce significantly higher oxidative stress and mitochondrial dysfunction.^76^ Specifically, weathered particles trigger a 1.5- to 2-fold increase in antioxidant enzyme markers (e.g., SOD, MDA) and cause lysosomal destabilization in marine organisms.^84^ Furthermore, the aging-induced porous structure facilitates the intracellular release of sequestered heavy metals, leading to inhibited ATP production and metabolic disruption.^77^^,^^78^

At the ecosystem level, biofilm complexes formed by aged plastics can alter community metabolic pathways, inducing measurable disturbances to local carbon and nitrogen cycles.^37^^,^^72^ In solid-phase environments, Gong et al. showed that the aging of biodegradable plastics alters soil aggregate structure and hydrological connectivity, affecting nutrient availability.^40^ Similarly, Li et al. pointed out that Fe-Natural organic matter (NOM) colloids can form “composite interfaces” with PET, either “locking” or “mobilizing” heavy metals at sediment-water interfaces.^25^ Yao et al. further noted that ROS hotspots drive the spatiotemporal heterogeneity of these interface reactions.^31^

However, risk intensity is context-dependent. Barhoumi et al. noted that PFAS adsorption on PS is strongly modulated by ionic strength,^18^ and Gao et al. reminded that laboratory weathering may not fully replicate real marine conditions.^7^ Xie et al. emphasized that the concentration and lifetime of EPFRs induced by aging vary greatly under different boundary conditions.^32^ Conversely, mitigation strategies are emerging: Sun et al. demonstrated that biochar can passivate metals while promoting plastic degradation,^79^ and Chen et al. proposed “gut microbiome intervention” for toxicity mitigation.^80^ Ultimately, aging generally increases interfacial reactivity and co-transport potential,^7^^,^^17^^,^^18^^,^^25^^,^^56^^,^^67^^,^^69^^,^^74^ but these effects are modulated by environmental boundary conditions and ecological processes.^25^^,^^35^^,^^79^^,^^81^

The abbreviations used throughout this article are summarized in Table 2.

**Table 2:** Abbreviations used in this article

Abbreviation	Full term
ARGs	Antibiotic resistance genes
DOM	Dissolved organic matter
EPFRs	Environmentally persistent free radicals
EPS	Extracellular polymeric substances
ETPU	Expanded thermoplastic polyurethane
FTIR	Fourier transform infrared spectroscopy
GPC	Gel permeation chromatography
HDPE	High-density polyethylene
HGT	Horizontal gene transfer
HPAM	Hydrolyzed polyacrylamide
LDPE	Low-density polyethylene
MPs	Microplastics
MWD	Molecular weight distribution
NOM	Natural organic matter
PA	Polyamide
PBAT	Polybutylene adipate terephthalate
PE	Polyethylene
PET	Polyethylene terephthalate
PFAS	Per- and poly-fluoroalkyl substances
PHB	Polyhydroxybutyrate
PLA	Polylactic acid
PP	Polypropylene
PS	Polystyrene
PVC	Polyvinyl chloride
PZC	Point of zero charge
ROS	Reactive oxygen species
SEM	Scanning electron microscopy
UV	Ultraviolet
XPS	X-ray photoelectron spectroscopy

## Conclusions and perspectives

### Conclusion

The review demonstrates that the aging of marine microplastics is effectively a process of uncontrolled materials engineering. By applying material science frameworks to environmental monitoring, the transition where microplastics shift from inert debris to active biological and chemical vectors can be better predicted. Aging of microplastics in marine environments is a complex process driven by the combined effects of light, oxidation, mechanical forces, and biological activity. The outcome of this process is the breaking of polymer molecular chains, changes in crystallinity and thermal properties, roughening of surface morphology, and accumulation of oxygen-containing functional groups. These changes increase the hydrophilicity and surface energy of microplastics, significantly enhancing their adsorption and co-transport capacity for organic pollutants, heavy metals, and ARGs. Meanwhile, the aging process promotes biofilm formation and microbial community succession, altering the migration and sedimentation pathways of microplastics in the environment, and potentially causing multidimensional risks to ecosystems and human health through the food chain. A review of existing research shows that the logical chain of aging, properties, behavior, and effects has been largely established. In contrast to previous reviews, a unified “interface reprogramming” framework is established to systematically link environmental drivers to structural responses. By shifting the focus from static descriptions to this dynamic mechanistic bridge, a theoretical foundation is provided for identifying the transition toward increased microplastic risk.

The unique contribution of this review lies in its “time-stamped” perspective, systematically linking aging drivers to surface evolution and environmental outcomes, which provides a dynamic framework for understanding microplastic risk that goes beyond static descriptions. These efforts will pave the way for a transition from mechanistic analysis to risk management, thereby providing a rigorous scientific foundation for science-based policy development and the implementation of effective mitigation measures. This “time-stamped” perspective is essential for supporting international plastic treaty negotiations and developing targeted mitigation strategies that address the true chemical and biological complexity of plastic pollution in the global ocean.

### Limitations of the study

However, there are still notable gaps in current research. First, most experiments remain under accelerated aging conditions in the laboratory, which differ from the multi-factor coupling in natural environments, leading to significant uncertainties when extrapolating results. Second, there is a lack of long-term *in situ* observations and multi-temporal data accumulation, making it difficult to systematically assess the dynamic changes in aging rates, mechanisms, and their environmental impacts. Moreover, there is insufficient research on the differences between materials (e.g., PP, PE, PLA, etc.) and environments (e.g., seawater, sediment, extreme environments, etc.), and a unified indicator system is lacking to ensure the comparability and integration of different studies. More critically, the interaction mechanisms between aged microplastics and EPFRs, ROS, and ARGs are still in the early stages, and quantification models and standardized evaluation frameworks for ecological risks have yet to be established.

Future research should focus on three directions: First, developing a multi-scale platform combining standardized aging experiments and *in situ* monitoring, and establishing comparable characterization indicators (e.g., carbonyl index, ζ potential, roughness, DOM release characteristics, etc.) to reduce the gap between experiments and natural environments. To ensure the comparability and integration of diverse studies, a tiered core indicator set is proposed as a universal benchmark for the field. At the primary level, chemical oxidation state must be defined using the CI and surface O/C ratios via XPS to quantify the baseline degradation of the polymer backbone. Furthermore, structural evolution descriptors, specifically specific surface area and MWD, are essential for predicting fragmentation kinetics and potential nanoplastic formation. Lastly, to characterize the particle’s role as a chemical and biological vector, interfacial reactivity indicators such as ζ potential and DOM release profiles must be reported to define the particle’s dynamic potential for pollutant adsorption. Adopting this prioritized core indicator set ensures that fragmented datasets can be synchronized into a unified, data-driven framework for predicting microplastic fate in the global ocean. Second, strengthening research on multi-factor interactions and specific scenarios, such as aging characteristics and their unique effects on ecological functions under extreme climate events, Fe-rich/S-rich environments, and saline-alkali waters. In specific marine niches, such as anaerobic sediments, different materials undergo distinct degradation routes. Therefore, future studies must clearly differentiate between the hydrolytic pathways of bio-based plastics (e.g., PLA and PBAT) and the photo-oxidative pathways of conventional polyolefins (e.g., PE, PP, etc.). Third, this review advocates for the transition from descriptive risk assessment to proactive mitigation based on interfacial engineering and biological intervention. Scientifically, this involves implementing “interfacial passivation” strategies, such as the use of functionalized biochar to simultaneously sequester heavy metals and accelerate microplastic mineralization, and developing optimized aerobic/anaerobic biological treatment protocols to intercept “pre-aged” particles at point sources. Additionally, “microbiome-mediated intervention,” including the regulation of gut microbiota to alleviate individual-level toxicity, offers a promising pathway for ecological recovery. Integrating these strategies with advanced predictive models, including surface-sensitive kinetic models to predict fragmentation, multiphase partition models to quantify pollutant co-transport, and machine learning-based QSAR models to assess toxicological risks under shifting climate scenarios. Furthermore, when applying machine learning approaches to fragmented environmental datasets, critical challenges such as high dimensionality and the risk of overfitting must be addressed to ensure model credibility. High dimensionality occurs when the number of input features, ranging from diverse polymer types to leached additive profiles, exceeds the available data points, often leading to models that capture noise rather than underlying physical laws. To mitigate these risks, rigorous model validation through techniques such as k-fold cross-validation and independent test sets is essential to assess generalizability. Additionally, sensitivity analysis and regularization are required to enhance predictive robustness and evaluate the impact of data uncertainties on model outcomes.

## Acknowledgments

This work was supported by the Key R&D Program of Jiangxi Province, China (20252BCF320020), and 10.13039/100008964Nanjing Normal University, China (184080H201B121).

## Author contributions

C.L., F.H., and M.X. contributed to conceptualization, review framework design, and overall manuscript organization. C.L., L.Z., J.L., Z.Z., and Z.C. contributed to literature search, literature screening, data extraction, evidence synthesis, and critical interpretation of the reviewed studies. C.L., L.Z., Z.Z., and Z.C. contributed to figure preparation, visualization, and refinement of the graphical presentation. C.L. wrote the original draft. L.Z., J.L., Z.Z., Z.C., D.Z., F.H., and M.X. contributed to manuscript revision, intellectual input, and writing – review and editing. D.Z., F.H., and M.X. provided supervision, resources, and project administration. F.H. and M.X. contributed to funding acquisition.

## Declaration of interests

The authors declare that they have no known competing financial interests or personal relationships that could have appeared to influence the work reported in this paper.
